# PREVALENCE of psychiatric morbidity in a community sample in Western Kenya

**DOI:** 10.1186/s12888-017-1202-9

**Published:** 2017-01-18

**Authors:** Edith Kwobah, Steve Epstein, Ann Mwangi, Debra Litzelman, Lukoye Atwoli

**Affiliations:** 1 Department of Mental Health, Moi University School of Medicine and Moi Teaching and Referral Hospital, PO Box 3, 30100 Eldoret, Kenya; 2 Department of Psychiatry, Georgetown University, 3800 reservoir Rd NW, Washington, DC 20007 USA; 3 Department of behavioral Sciences, Moi University School of Medicine, P.O Box 4606, Eldoret, Kenya; 4 Department of Medicine, Indiana University School of medicine, 002 Wishard Blvd 4, Indianapolis, IN 46202 USA; 5 Department of Mental Health, Moi University School of Medicine, P.O Box 4606, Eldoret, Kenya

**Keywords:** Mental disorders, Prevalence, Mini International Neuropsychiatry Interview, Western Kenya

## Abstract

**Background:**

About 25% of the worldwide population suffers from mental, neurological and substance use disorders but unfortunately, up to 75% of affected persons do not have access to the treatment they need. Data on the magnitude of the mental health problem in Kenya is scarce.

The objectives of this study were to establish the prevalence and the socio-demographic factors associated with mental and substance use disorders in Kosirai division, Nandi County, Western Kenya.

**Methods:**

This was a cross sectional descriptive study in which participants were selected by simple random sampling. The sampling frame was obtained from a data base of the population in the study area developed during door-to-door testing and counseling exercises for HIV/AIDS. Four hundred and twenty consenting adults were interviewed by psychologists using the Mini International Neuropsychiatric Interview Version 7 for Diagnostic and Statistical Manual 5th Edition and a researcher-designed social demographic questionnaire.

**Results:**

One hundred and ninety one (45%) of the participants had a lifetime diagnosis of at least one of the mental disorders. Of these, 66 (15.7%) had anxiety disorder, 53 (12.3%) had major depressive disorder; 49 (11.7%) had alcohol and substance use disorder. 32 (7.6%) had experienced a psychotic episode and 69 (16.4%) had a life-time suicidal attempt. Only 7 (1.7%) had ever been diagnosed with a mental illness. Having a mental condition was associated with age less than 60 years and having a medical condition.

**Conclusion:**

A large proportion of the community has had a mental disorder in their lifetime and most of these conditions are undiagnosed and therefore not treated. These findings indicate a need for strategies that will promote diagnosis and treatment of community members with psychiatric disorders. In order to screen more people for mental illness, we recommend further research to evaluate a strategy similar to the home based counseling and testing for HIV and the use of simple screening tools.

## Background

About 25% of the population suffers from Mental, Neurological and Substance use disorders (MNS) and 14% of the global burden of disease is attributed to these disorders, but up to 75% of affected persons in many low and middle income countries do not have access to the treatment they need [1]. The objectives of the WHO mental health action plan 2013–2020 include provision of comprehensive, integrated and responsive mental health care services in community-based settings, and also emphasize the empowerment of people with mental disabilities, the need to develop a strong civil society and the importance of mental health promotion and prevention [2].

There are limited resources for mental health in many Low and Middle Income Countries (LMICs), with less than 1% of the total health budget being allocated for mental health and most of the available resources are directed towards treating severely ill patients in major psychiatric hospitals [3, 4] In many LMICs, human resources for mental health are also scarce and generally inaccessible. In Kenya for example, there are fewer than 100 psychiatrists, [5] most of who are based in Universities, National Referral Hospitals and a few county hospitals. Data on the burden of mental disorders at the community level are limited. Most of the existing data in Kenya today are based on research conducted in hospital settings, which may not be a true reflection of the status at the community level. One population based survey done in Nyanza revealed a 10.3% prevalence rate of common mental disorders [6].

The aim of this study was to generate evidence on the burden of mental disorders in a community sample in western Kenya. The findings of this study should provide an evidence base for the development of community based solutions to the huge treatment gap in western Kenya, and inform relevant policies that will then enable the integration of mental health services into primary care facilities.

## Methods

### Study design

This was a cross sectional descriptive study.

### Setting

The study was conducted in Kosirai Division, Nandi County in the western part of Kenya. The study area is inhabited by approximately 36,000 people across 6,000 households, organized in 100 villages [7]. Like the rest of Nandi County, and indeed the rest of western Kenya, this community is predominantly rural with farming as the leading economic activity. This area serves as the catchment area of Mosoriot sub-county hospital, one of the sites in the AMPATH (Academic Model Providing Access to Healthcare) coverage. AMPATH is a consortium of Moi University, Moi Teaching and Referral Hospital, and North American Universities, with an existing clinical care delivery system and research infrastructure for HIV, hypertension, diabetes and cervical cancer [8]. The initial plans to integrate mental health in the AMPATH systems are underway. Kosirai division has a primary care facility staffed with a psychiatric nurse which makes it a place to start evidence based mental health work which can be scaled up to other AMPATH sites. This study forms part of the foundation on which these plans will be built.

### Study population and sampling

The study targeted adults living in Kosirai Division in the period January 2014 – November 2015. Participants were selected by simple random sampling from a data base that was developed during door to door HIV/Aids counseling exercises targeting the entire population in the study area. Demographic data of persons above 14 years of age was captured regardless of the outcome of the HIV test. Published work describing the door to door strategy indicate that up to 97% of the households in the study area were accessed during this exercise [9] hence giving confidence to the authors that such data is fairly representative of the target population. From the database 8000 individuals aged 18 years and above were included in the sampling frame. A sample size of 384 participants was calculated using the Cochran formulae for sample size determination, *n* = Z^2^pq/e. [10]. Given the complexity of tracing individuals in their homesteads we increased the sample size by 50%, to adjust for those who may not be traced and avoid resampling and replacements. A total of 570 participants were sampled out of who 450 were traced. 25 persons did not meet the inclusion criteria: either they were younger than 18 years or they could not the interview languages, speak Kiswahili or English and five eligible participants did not give consent to participate. A total of 420 participants were therefore interviewed for this study.

### Outcome measures

Socio-demographic characteristics including age, gender, marital status and education level were collected using a researcher-designed questionnaire.

Psychiatric morbidity was defined as having at least one of the disorders included in the Mini International Neuropsychiatric Interview (MINI) Version 7 for Diagnostic and Statistical Manual 5th Edition (DSM-5). MINI is a short diagnostic structured interview, developed for Axis I psychiatric disorders and it has been shown to be reliable compared to the Structured Clinical Interview for DSM (SCID) and the Composite International Diagnostic Interview (CIDI), but with a shorter administration time [11]. It has been used in Kenya in a study by Ndetei et al. after adaptation and adoption [12]. For this study, a written permission to use and translate the MINI was granted by the author, Dr. Sheehan.

### Study procedures

Data were collected from December 2015 to January 2016 by two assistants with masters’ level training in psychology. The psychologists underwent a 5 day study-specific training conducted by a psychiatrist. The training involved understanding the content of the MINI, coding for various diagnoses, assessment of various symptoms, socio-demographic questionnaire and the consenting process. They carried out practical interviews with healthy adults and with stable mentally ill patients admitted in a mental health ward. The diagnosis from the MINI interviews were compared with the clinical interviews done by a psychiatrist and were rated as satisfactory.

Community health volunteers embedded in the study community mobilized the sampled community members for the study prior to the data collection process. To facilitate community entry, the research assistants were accompanied by community health volunteers into the homesteads during the interviews. The interviews were conducted in the homesteads in the most private place and the participant was interviewed alone except in cases where corroborative history was needed.

### Data and analysis

Data were collected on paper and later entered into a password protected database while the original paper forms were stored in a locked cabinet. After data entry was completed, data were cleaned and analyzed using the STATA version 13 [13]. Descriptive statistics were used for continuous data and frequency listings were used for categorical variables. Chi square tests were used to assess factors associated with prevalence of mental disorders. All analyses were carried out at 95% level of significance.

## Results

A total of four hundred and twenty adults participated in the survey. The participants had a median age of 34 years and an interquartile range of 27–46 years with an almost equal male to female ratio. The study population was of a relatively low social economic background given that most of the participants earned less than 10 000 Kenya shillings (100 USD) per month. Very few participants reported ever been diagnosed or treated for mental illness (See Table 1).

**Table 1:** Social demographic characteristics of the participants

Variable		Frequency *N* = 420	Percentage
Sex	Male	204	48.6
	Female	216	51.4
Marital status	Married	258	61.4
	Single	137	32.6
	Widowed/Divorced/Separated	25	6.0
Religion	Muslim	8	1.9
	Christian	405	96.7
	Buddhist	2	0.5
	Others	4	1.0
Education level	None/Primary	194	46.2
	Secondary/Post-Secondary	226	53.8
Employment	Unemployed/Casual	127	30.2
	Formal employment	50	11.9
	Self-employment	243	57.9
Income	<10000	271	64.5
	>10 000	149	35.5
Have a medical condition	No	349	83.1
	Yes	71	16.9
Ever been diagnosed with mental illness	Yes	413	98.3
	No	7	1.7

Lifetime prevalence of any DSM-5 mental disorder as measured by the MINI-7 was 45.5% in this sample. Only 3.6% of those who screened positive for mental illness (7/191) had ever been diagnosed with mental illness.

As shown on Table 2 anxiety disorders, major depression and alcohol use disorders had the highest prevalence rates. Only 1.7% (7/ 420) had ever had an eating disorder. While psychotic episodes and suicidal attempts are not standalone diagnosis on DSM V, the high rates in this rural community (7.6% and 16.4% respectively) are worth highlighting.

**Table 2:** Lifetime Prevalence of mental disorders as described in the MINI 7 for DSM V

Disorder	Actual number	(%)
Any mental disorder	191	45.5
Anxiety disorders	66	15.7
Major Depressive Disorder	53	12.6
Alcohol/substance use disorders	49	11.7
Suicide behavior disorder	28	6.7
Bipolar mood disorder	22	5.2
PTSD	19	4.5
Psychotic Disorder	4	1.0
Antisocial Personality Disorder	13	3.1
Eating disorders	7	1.7
Other significant conditions		
Suicidal attempt	64	16.4
Psychotic episodes	32	7.6

In the correlation analysis shown on Table 3, the lifetime prevalence of any mental disorder was significantly associated with having a medical condition and age. In the logistic regression the significant variables were medical condition and age above 60 years. Adjusting for gender, marital status, education, employment and medical condition patients aged above 60 years had a lower odds (OR = 0.26, 95% CI 0.09–0.74) of having a mental disorder compared to those below 30 years. Adjusting for age, gender, marital status, education and employment in patients with a medical condition, the odds of having a mental disorder was 2.5 times that of those without a medical condition (O.R 2.53; 95% CI 1.44–4.43 as illustrated on Table 4. None of the other socio-demographic factors showed any association with lifetime mental disorder**.**

**Table 3:** Correlation analysis of any mental disorder and socio-demographic characteristics

	Lifetime Mental Disorder		Chi-square
Age in categories	Absent	Present	*p*-value
<30	82(53.2)	72(46.8)	0.038
30–60	122(52.4)	111(47.6)	
>60	25(75.8)	8(24.2)	
Sex			
Male	116(56.9)	88(43.1)	0.35
Female	113(52.3)	103(47.7)	
Marital status			
Married	149(57.8)	109(42.2)	0.145
Single	70 (51.1)	67 (48.9)	
Widowed/divorce/separated	10(40.0)	15(60.0)	
Education level			
None/Primary	99(51)	95(49)	0.183
Post primary	130(57.5)	96(42.5)	
Employment			
Unemployed	61(48)	66(52)	0.186
Formal	27(54)	23(46)	
Self employed	141(58)	102(42)	
Income			
<10,000	147(54.2)	124(45.8)	0.876
>10,000	82(55)	67(45)	
Medical condition			
Absent	204(58.5)	145(41.5)	<0.0001
Present	25(35.2)	46(64.8)

**Table 4:** Multiple logistic regression model of the association between any mental disorder and socio-demographic characteristics

Variable	Adjusted Odds Ratio	*p*-value	[95% Conf. Interval]	
30–60 vs <30 years	1.07	0.79	0.64	1.78
>60 vs <30 years	0.26	0.01	0.09	0.74
Female vs Male	1.02	0.93	0.68	1.54
Single vs Married	1.28	0.34	0.77	2.11
(Widowed/Divorced/Separated) vs Married	2.22	0.11	0.83	5.95
Postprimary vs Primary/No education	0.69	0.10	0.45	1.08
Formal vs Unemployment	0.85	0.65	0.42	1.72
Self-employed vs Unemployed	0.72	0.21	0.43	1.20
Medical condition (Present vs Absent)	2.53	0.00	1.44	4.43

## Discussion

This study makes an important contribution, reporting for the first time the prevalence and factors associated with DSM-5 mental disorders in a rural community in an LMIC setting in sub-Saharan Africa. The prevalence rate we report of 45.5% is significantly higher than what most previous studies have found. One possible reason for the differences in the diagnostic tools used in the different studies. In this study, we used the MINI version 7 for DSM-5, which captures suicidal behavior disorder and also includes personality disorders apart from other severe mental disorders. For example, a population based household survey carried out in 2013 in a demographic surveillance site in Kisumu in western Kenya found that the overall prevalence of Common Mental Disorders was 10.3% using the Clinical Schedule Revised (CSR) [6]. This study also found that depression and anxiety were the most common disorders similar to our study where anxiety, depression and substance use disorders were the most common diagnoses. Further, a meta-analysis that included studies conducted between 1980 and 2013 around the globe reported lower lifetime prevalence of mental disorders than what we report in this study, ranging from 17.6% to 29.2% [14]. In a Nigerian survey involving 4984 people it was reported that 12.1% of the participants had a lifetime rate of at least one DSM-IV disorder with anxiety disorders being most common [15]. Finally, the South African Stress and Health Survey (SASH) found a lifetime prevalence rate for any mental disorder to be 30.3%, using the WHO World Mental Health Survey’s Composite International Diagnostic Interview (CIDI) for DSM IV. Similar to our findings, the most prevalent lifetime disorders in the SASH survey were anxiety disorders [16].

In the present study, despite many participants having experienced some mental distress in their lifetime, only 3.6% (7 out of 191 who screened positive) had ever received care, indicating that a majority of people suffering mental illness in this region remained undiagnosed and untreated. Huge treatment gaps have been described in most parts of the world as demonstrated in a study involving Netherlands, Chile, United States, Canada and Germany that found that up to 60% of persons with severe mental disorders were not receiving treatment [17]. A study by Ndetei and colleagues also found that about 25% of patients in general hospital settings have mental disorders that go unrecognized and are often undertreated even when correctly diagnosed [18].

Age was associated with lower rates of having a lifetime mental disorders, with respondents aged over 60 years having rates of 24.2% compared to those younger than 60 years who had rates above 45%. It is difficult to explain the lower prevalence of mental disorders among older adults compared to those less than 60 years of age, but their relatively small proportion in this sample might be responsible for the difference. Persons who reported having a medical condition had a lifetime prevalence of mental disorder of 64.8% compared to 41.5% among those who did not report a medical condition. The relationship between mental illness and various medical conditions has been widely described in literature. There is evidence of increased mental disorders among patients suffering from infectious diseases like tuberculosis, HIV and also among non-communicable diseases like diabetes, hypertension and cancer [19–23]. Further, it has also been shown that persons suffering from mental disorders have a greater burden of chronic medical conditions than the general population [24]. In this study, we did not study the temporal association between the medical conditions and mental disorders, and further work, including longitudinal studies, may better describe the relationships.

Although this study did not demonstrate gender association with a lifetime mental disorder, studies have demonstrated gender differences in the prevalence of common mental disorder with women having higher rates of mood and anxiety disorders and men having higher rates of substance use disorders [25]. Similar findings were shown in a face to face household survey by WHO which aimed to establish variation in gender differences in lifetime DSM-IV mental disorders across cohorts in 15 countries in the World Health Organization World Mental Health Survey. In that study, women had more anxiety and mood disorders than men, and men had more externalizing and substance disorders than women [26]. The study in Nyanza Kenya showed significantly higher rates of having any CMD in 2013 among women [6].

None of the other socio-demographic factors were associated with a lifetime mental disorder diagnosis, or any specific disorder in this study. This is contrary to findings in other community surveys showing association with marital status [16, 27], education level [28, 29] and socio-economic status [30]. A follow up study with a larger sample size in this area would be useful to explore these possible associations as well as the reasons for lack of association with factors found to increase risk elsewhere.

### Study limitations

A number of important limitations must be appreciated in understanding findings in this study. Firstly, diagnostic errors are possible especially in the section assessing for psychosis because it required interviewer observation and judgment. Secondly, a non- reporting bias is possible especially because of fear of being labeled mentally ill in a setting in which mental illness is highly stigmatized. Another limitation is that the specific medical disorders were not assessed.

## Conclusion

A large proportion of the community has mental disorders and most of these conditions are undiagnosed and therefore not treated. These findings indicate a need to have strategies that will promote diagnosis and treatment of community members with psychiatric disorders. They also indicate a need for mental healthcare services at the community level in order to ensure accessibility of affordable services. In order to screen more people for mental illness, we recommend further research to evaluate a strategy similar to the home based counselling and testing for HIV and the use of simple screening tools.

## Abbreviations

AMPATH: Academic model providing access to health care
CIDI: Composite international diagnostic interview
CMD: Common mental disorder
DSM: Diagnostic statistical manual
MINI: Mini international neuropsychiatric interview
MNS: Mental, neurological and substance use disorders
SCID: Structured clinical interview for DSM III disorders
WHO: World Health Organization.
